# Dental visits in Medicaid-enrolled youth with mental illness: an analysis of administrative claims data

**DOI:** 10.1186/s12913-020-05973-1

**Published:** 2020-12-11

**Authors:** Erica L. Stockbridge, Eleena Dhakal, Stacey B. Griner, Abiah D. Loethen, Joseph F. West, Joseph W. Vera, Karabi Nandy

**Affiliations:** 1 Department of Health Behavior & Health Systems, School of Public Health, University of North Texas Health Science Center, 3500 Camp Bowie Blvd, Fort Worth, TX 76107 USA; 2 Department of Advanced Health Analytics and Solutions, Magellan Health, Inc., 4800 N Scottsdale Rd #4400, Scottsdale, AZ 85251 USA; 3 Department of Biostatistics and Epidemiology, School of Public Health, University of North Texas Health Science Center, 3500 Camp Bowie Blvd, Fort Worth, TX 76107 USA; 4 Florida Institute for Health Innovation, 2701 N. Australian Avenue Suite 204, West Palm Beach, Florida, 33407 USA; 5 Department of Public Health Sciences, Miller School of Medicine, University of Miami, 1600 NW 10th Ave #1140, Miami, Florida 33136 USA; 6 Department of Population and Data Sciences, UT Southwestern Medical Center, South Campus, 5323 Harry Hines Blvd, Dallas, TX 75390 USA

**Keywords:** Oral health, Dental care, Healthcare utilization, Mental illness, Healthcare effectiveness data and information set (HEDIS), Annual dental visits, Medicaid, Social determinants of health, Health services research, Claims data, Children, Adolescents, Youth

## Abstract

**Background:**

State Medicaid plans across the United States provide dental insurance coverage to millions of young persons with mental illness (MI), including those with attention deficit hyperactivity disorder (ADHD), depression, anxiety, bipolar disorder, and schizophrenia. There are significant oral health challenges associated with MI, and providing dental care to persons with MI while they are young provides a foundation for future oral health. However, little is known about the factors associated with the receipt of dental care in young Medicaid enrollees with MI. We aimed to identify mental and physical health and sociodemographic characteristics associated with dental visits among this population.

**Methods:**

We retrospectively analyzed administrative claims data from a Medicaid specialty health plan (September 2014 to December 2015). All enrollees in the plan had MI and were ≥ 7 years of age; data for enrollees aged 7 to 20 years were analyzed. We used two-level, mixed effects regression models to explore the relationships between enrollee characteristics and dental visits during 2015.

**Results:**

Of 6564 Medicaid-enrolled youth with MI, 29.0% (95% CI, 27.9, 30.1%) had one or more visits with a dentist or dental hygienist. Within youth with MI, neither anxiety (Adjusted odds ratio [AOR] = 1.15, *p* = 0.111), post-traumatic stress disorder (AOR = 1.31, *p* = 0.075), depression (AOR = 1.02, *p* = 0.831), bipolar disorder (AOR = 0.97, *p* = 0.759), nor schizophrenia (AOR = 0.83, *p* = 0.199) was associated with dental visits in adjusted analyses, although having ADHD was significantly associated with higher odds of dental visits relative to not having this condition (AOR = 1.34, *p* < 0.001). Age, sex, race/ethnicity, language, and education were also significantly associated with visits (*p* < 0.05 for all).

**Conclusions:**

Dental utilization as measured by annual dental visits was lower in Medicaid-enrolled youth with MI relative to the general population of Medicaid-enrolled youth. However, utilization varied within the population of Medicaid-enrolled youth with MI, and we identified a number of characteristics significantly associated with the receipt of dental services. By identifying these variations in dental service use this study facilitates the development of targeted strategies to increase the use of dental care in – and consequently improve the current and long-term wellbeing of – the vulnerable population of Medicaid-enrolled youth with MI.

## Background

There is an increasing recognition of the association between mental health conditions and oral health [1–4]. Relative to persons without mental illness (MI), persons with MI are more likely to experience dental decay, periodontal disease, and tooth loss [3, 4]. Risk factors including poor nutritional status, poor oral hygiene, high intake of sugary drinks, and low socioeconomic status are more prevalent in persons with MI [1, 3]. Further, many psychotropic medications used for mental health treatment have nontrivial side effects that affect oral health (e.g., reduction in salivary flow which can lead to increases in xerostomia and increased susceptibility to dental caries) [5]. Consequently, dental care in persons with MI is critical to both prevent the development of new oral health issues and ensure the timely treatment of incipient conditions.

Providing dental care to persons with MI while they are young provides a foundation for future establishment of a dental home and oral health. The increasing prevalence of mental health issues among children and adolescents [6] suggests that there are increasing opportunities for proactive oral health-focused interventions in this population. MI is of particular concern for older children and adolescents because the prevalence of MI in children and adolescents increases with age; 13 to 20% of US children have some form of mental health disorder [7] and approximately one in four to five adolescents in the US meet the criteria for a mental health disorder [8]. Adolescence is also when many serious MIs (e.g., major depression, bipolar disorder, and schizophrenia) begin to emerge [9]. At the same time, MI in young persons may be exacerbated in the presence of poor oral health, as poor oral health can negatively affect socialization, psychological well-being, behavior, and self-esteem [10, 11]. Thus, there is a need for population health management programs to focus on the use of dental services in older children and adolescents with MI.

In the US, all state Medicaid plans are required to cover dental services for enrollees under age 21 years and 14% of young persons in Medicaid have a diagnosed behavioral health condition [12]. Thus, millions of youth with MI have dental insurance coverage through Medicaid. Given the significant oral health challenges associated with MI, it is important to facilitate dental visits in this subgroup of Medicaid enrollees. Although it has been identified that Medicaid-enrolled children who qualified for Medicaid due to disability are less likely to have annual dental visits than those qualified based on income [13], we identified no studies which examined the factors associated with dental visits in Medicaid-enrolled youth with MI. Understanding this vulnerable population’s utilization of dental services is necessary for state Medicaid agencies, Medicaid managed care programs, dental benefit management organizations, and population health management programs to develop targeted strategies to increase the use of dental care. Our exploratory study aimed to support those efforts by identifying the mental and physical health and sociodemographic characteristics associated with dental visits in Medicaid-enrolled youth with MI.

## Methods

The Office of Research Compliance at the University of North Texas Health Science Center determined on behalf of the North Texas Regional Institutional Review Board that this work is not human subjects research.

### Data source & study population

Administrative data from the Magellan Complete Care (MCC) of Florida Medicaid health plan were used for this study. During the period studied, MCC operated in Florida Medicaid regions 2, 4, 5, 6, 7, 9, 10, and 11, encompassing 40 of Florida’s 67 counties. The regions with the most MCC of Florida enrollees were region 11 (Miami-Dade and Monroe counties) and region 6 (Hardee, Highlands, Hillsborough, Manatee and Polk counties). As of December 2015, 42,138 Medicaid enrollees in the state of Florida were enrolled in this plan; 31% were ages 7 to 20 years.

MCC of Florida was the first Medicaid specialty plan designed specifically to support the complex biopsychosocial needs of persons with serious MI. State Medicaid programs are increasingly adopting specialty plans for high-needs populations, although they are not mandated by the Centers for Medicare and Medicaid Services [14, 15]. The MCC of Florida plan was available to persons in Florida ages 7 and older with serious MI; those less than 7 years of age and those without serious MI were not eligible for enrollment. The Florida Agency for Health Care Administration determined mental health status for the purposes of plan eligibility based on claims data collected prior to enrollment. Eligible individuals were those with a prior diagnosis of major depression, bipolar disorder, obsessive-compulsive disorder, schizophrenia, or another psychotic or delusional disorder, or the person had previously received a prescription for a medication used to treat these disorders. Florida Medicaid beneficiaries meeting these criteria were automatically enrolled in this plan unless the member opted otherwise [16]. Given these requirements, all individuals included our analyses had a prior MI diagnosis or medication. MCC of Florida enrollees were included in this study if they were 7 to 20 years of age and had continuous enrollment (i.e., no more than one gap of up to 45 days) during 2015. All such enrollees had Medicaid dental benefits. Enrollment data and dental, medical, and pharmacy claims data from services rendered September 2014 through December 2015 were combined for analysis.

### Measures

#### Outcome variable

The outcome of interest was whether an enrollee had one or more dental visits with a dental practitioner during 2015. Dental visits were identified using procedure codes in claims data, consistent with the National Committee for Quality Assurance (NCQA) Healthcare Effectiveness Data and Information Set (HEDIS) Annual Dental Visit quality measure for measurement year 2015 [17]. The Annual Dental Visit procedure code set included American Dental Association’s Codes on Dental Procedures and Nomenclature (CDT) for both preventive and treatment services, and it included Current Procedural Terminology (CPT) codes for dental radiologic exams. Providers were deemed to be dental practitioners if they held a Doctor of Dental Surgery (DDS) or a Doctor of Dental Medicine (DMD) from an accredited institute and were licensed in their state of practice. Dental practitioners also included certified and licensed dental hygienists, as did specialists meeting the degree and licensure requirements (e.g., pediatric dentists, orthodontists) [17].

#### Explanatory variables

We selected explanatory variables based on Andersen’s Model of Health Services Use, which proposed that the use of healthcare services is driven by clinical need (e.g., health conditions), enabling resources (e.g., affordability, availability of providers, health insurance), and predisposing characteristics (e.g., sex, age) [18]. We extracted most predisposing characteristic variables from health plan enrollment data, including age, sex, race/ethnicity, and Medicaid eligibility group (Supplemental Security Income [SSI], Temporary Assistance for Needy Families [TANF], or Other). We grouped ages based on HEDIS categorizations and included the education levels of adults in each enrollee’s county of residence. A dichotomous variable represented whether enrollees had any claims for non-dental health services during 2015.

Enabling resource variables were either extracted from enrollment data or generated based on enrollees’ counties of residence. These variables included language (not English speaker/English speaker) and county classification (urban/rural). We also considered Health Resources and Services Administration geographic health professional shortage area (HPSA) designations [19] . Specifically, we included variables indicating whether the county was a designated geographic health professional shortage area for primary care physicians (PCP HPSA) and/or mental health professionals (MH HPSA). No members’ counties were designated geographic dental health professional shortage areas so this variable was excluded.

Clinical need was represented by health condition variables generated from claims for services rendered between September 2014 and December 2015. Optum Impact Pro clinical indicators generated from diagnoses documented in claims data were used to identify health conditions [20]. The mental health conditions we examined were attention deficit hyperactivity disorder (ADHD), depression, bipolar disorder, schizophrenia, post-traumatic stress disorder (PTSD), and non-PTSD anxiety disorder (including obsessive-compulsive disorder). We also examined high-prevalence comorbid conditions in young persons with MI, including asthma, diabetes, hypertension, pregnancy, and substance use disorder (encompassing both alcohol and drug use disorders). Finally, as a measure of overall healthcare need, we included a prospective risk score generated by a predictive regression model within the Impact Pro software. To generate this score, Impact Pro first assigns the individual healthcare services represented in claims data into patient-level clinical episodes of care representing different courses of treatment for health conditions. The software then uses a mathematical function to link the patient-level episode data and other administrative data to plan members’ predicted future healthcare costs for the subsequent 12 months. A score of 1.0 represented average risk (i.e., average future healthcare costs were predicted), while scores above or below 1.0 represented higher or lower risk, respectively [20].

### Statistical methods

We conducted all statistical analyses with two-level, mixed effects regression models that controlled for members’ primary care physicians as a random effect. We examined unadjusted relationships using bivariate models, and then used two multivariable mixed effects logistic regression models to estimate the adjusted associations between the likelihood of a dental visit and the explanatory variables. Model 1 examined predisposing and enabling variables but excluded need variables; all individuals meeting study criteria were included. Model 2 included all variables. Because need variables were constructed based on claims data for non-dental healthcare services, Model 2 excluded persons who had no claims for non-dental services. Additionally, we ran bivariate post hoc models on persons included in Model 2 to examine the associations between substance use disorder and other variables of interest.

We used the multivariable models to calculate the average predicted probabilities of dental visits for each level of the explanatory variables, and we generated a graph to illustrate the relationship between the probabilities and prospective risk score (our only continuous variable). We used Stata 14.1 [StataCorp, College Station, TX] to conduct statistical testing; testing was two-sided with significance tested at *p* < 0.05.

## Results

Of 6564 Medicaid-enrolled youth with MI, 29.0% (*n* = 1904) had one or more dental visits during 2015. Table 1 describes sample characteristics in total and by dental visit status. The most commonly diagnosed mental health condition was ADHD (29.6%). Additionally, 23.6% experienced depression, 17.8% experienced bipolar disorder, 15.1% experienced anxiety disorder, 5.4% experienced schizophrenia, and 4.1% experienced PTSD. Percentages do not sum to 100% because some youth received multiple MI diagnoses while others had no healthcare claims with MI diagnoses during the period studied. Of the youth studied, 88.4% (*n* = 5805) had claims for non-dental healthcare services.

**Table 1:** Characteristics of Medicaid-enrolled youth with mental illness, overall and by 2015 dental visit status. Based on health plan enrollment data and medical, pharmacy, and dental claims data with service dates between September 2014 and December 2015 (*n* = 6564 for sociodemographic variables; *n* = 5805 for health condition and prospective risk score variables)

Enrollee Characteristics	Overall n (%) or mean (SD)	One or more dental visits during the year n (%) or mean (SD)	No dental visits during the year n (%) or mean (SD)	*p*-value
Sex				
Female	3301 (50.29)	1011 (53.10)	2290 (49.14)	0.004
Male	3263 (49.71)	893 (46.90)	2370 (50.86)	
Age Group				
7–10	630 (9.60)	247 (12.97)	383 (8.22)	< 0.001
11–14	1540 (23.46)	503 (26.42)	1037 (22.25)	
15–18	3277 (49.92)	939 (49.32)	2338 (50.17)	
19–20	1117 (17.02)	215 (11.29)	902 (19.36)	
Race/Ethnicity				
White	1881 (28.66)	510 (26.79)	1371 (29.42)	0.001
Black / African American	1826 (27.82)	496 (26.05)	1330 (28.54)	
Hispanic	1379 (21.01)	448 (23.53)	931 (19.98)	
Other	182 (2.77)	52 (2.73)	130 (2.79)	
Not Provided	1296 (19.74)	398 (20.90)	898 (19.27)	
Language				
English	5858 (89.24)	16.51 (86.71)	4207 (90.28)	< 0.001
Not English	706 (10.76)	253 (13.29)	453 (9.72)	
Education Levels in County				
15% + of adults have HS degree	4650 (70.84)	1397 (73.37)	3253 (69.81)	0.014
< 15% of adults have HS degree	1914 (29.16)	507 (26.63)	1407 (30.19)	
Medicaid Eligibility Group				
SSI	1830 (27.88)	540 (28.36)	1290 (27.68)	0.754
TANF	4552 (69.35)	1310 (68.80)	3242 (69.57)	
Other	182 (2.77)	54 (2.84)		
NCHS Urban-Rural Category				
Large Central Metro (Most Urban)	2922 (44.52)	836 (43.91)	2086 (44.76)	0.114
Large Fringe Metro	1680 (25.59)	532 (27.94)	1148 (24.64)	
Medium Metro	1571 (23.93)	419 (22.01)	1152 (24.72)	
Small Metro	178 (2.71)	52 (2.73)	126 (2.70)	
Non- Metro (Most Rural)	213 (3.24)	65 (3.41)	148 (3.18)	
County is PCP HPSA				
No	6465 (98.49)	1884 (98.95)	4581 (98.30)	0.167
Yes	99 (1.51)	20 (1.05)	79 (1.70)	
County is MH HPSA				
No	5804 (88.42)	1686 (88.55)	4118 (88.37)	0.743
Yes	760 (11.58)	218 (11.45)	542 (11.63)	
Did Member Have any Non-Dental Claims?				
No	759 (11.56)	96 (5.04)	663 (14.230	< 0.001
Yes	5805 (88.44)	1808 (94.96)	3997 (85.77)	
ADHD				
No	4085 (70.37)	1164 (64.38)	2921 (73.08)	< 0.001
Yes	1720 (29.63)	644 (35.62)	1076 (26.92)	
Depression				
No	4437 (76.73)	1359 (75.17)	3078 (77.01)	0.117
Yes	1368 (23.57)	449 (24.83)	919 (22.99)	
Bipolar				
No	4771 (82.191)	1480 (81.86)	3291 (82.34)	0.544
Yes	1034 (17.81)	328 (18.14)	706 (17.66)	
Schizophrenia				
No	5489 (94.56)	1725 (95.41)	3764 (94.17)	0.063
Yes	316 (5.44)	83 (4.59)	233 (5.83)	
PTSD				
No	5570 (95.95)	1717 (94.97)	3853 (96.40)	0.010
Yes	235 (4.05)	91 (5.03)	144 (3.60)	
Anxiety, no PTSD				
No	4927 (84.88)	1510 (83.52)	3417 (85.49)	0.052
Yes	878 (15.12)	298 (16.48)	580 (14.91)	
Asthma				
No	4687(80.74)	1419 (78.48)	3268 (81.76)	0.004
Yes	1118 (19.26)	381(21.52)	729 (18.24)	
Diabetes				
No	5698 (98.16)	1772 (98.01)	3926 (98.22)	0.599
Yes	107 (1.84)	36 (1.99)	71 (1.78)	
Hypertension				
No	4862 (83.76)	1460 (80.75)	3402 (85.11)	< 0.001
Yes	943 (16.24)	348 (19.25)	595 (14.89)	
Pregnancy				
No	5515 (95.0)	1746 (96.57)	3769 (94.30)	< 0.001
Yes	290 (5.0)	62 (3.43)	228 (5.70)	
Prospective Risk Score				
*(Continuous)*	1.09 (1.25)	1.16 (1.24)	1.06 (1.25)	0.004
Substance Use Disorder				
No	5055 (87.08)	1602 (88.61)	3453 (86.39)	0.026
Yes	750 (12.92)	206 (11.39)	544 (13.61)	

*Abbreviations*: *ADHD* Attention deficit/hyperactivity disorder, *HPSA* Health professional shortage area, *HS* High school, *NCHS* National Center for Health Statistics, *MH* Mental Health, *PCP* Primary care provider, *PTSD* Post-traumatic stress disorder, *SSI* Supplemental Security Income, *TANF* Temporary Aid for Needy Families

**Fig. 1:**
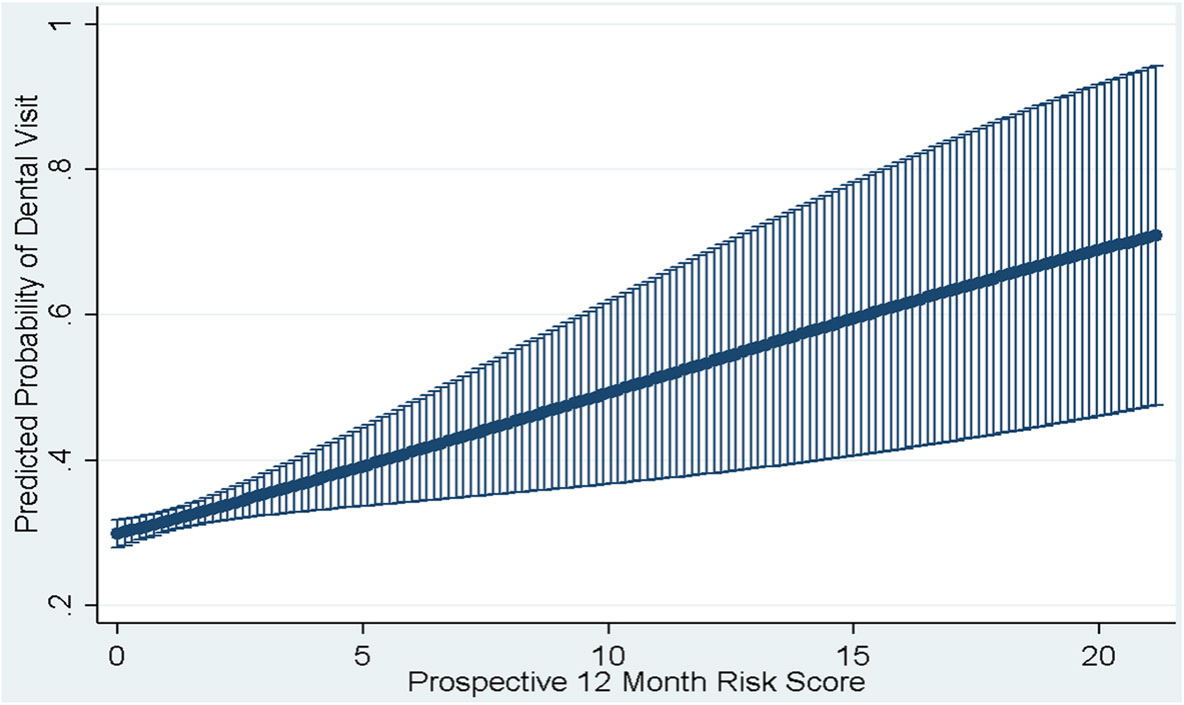
Average predicted probabilities of having one or more dental visits by prospective risk score, based on logistic regression model results examining the likelihood of Medicaid-enrolled youth with mental illness having one or more dental visits during 2015 (*n* = 5805)

The unadjusted associations between the characteristics of the youth and the receipt of one or more dental visits are reported in Table 1, including *p*-values and 95% confidence intervals. Table 2 provides details regarding the adjusted associations between the characteristics of the youth and the receipt of one or more dental visits, including odds ratios, 95% confidence intervals, and p-values. Average predicted probabilities for all categorical variables are also provided in Table 2 and average predicted probabilities based on prospective risk scores are illustrated in Fig. 1. Results for sociodemographic variables and the variable representing the presence/absence of non-dental claims are based on the entire sample (*n* = 6564), while results for health condition variables and prospective risk score are based on the *n* = 5805 youth with claims data. Youth with claims had significantly greater odds of dental visits compared to those without (Table 2, Model 1). Even so, findings were similar for variables included in both multivariable Models 1 and 2 which included the full sample and the restricted sample, respectively. Further, likelihood ratio tests suggested that both models fit the data well (Model 1 X^2^ = 11.86, *p*-value = 0.0003; Model 2 X^2^ = 14.58, *p*-value = 0.0001). Accordingly, the adjusted models are interpreted together.

**Table 2:** Results of two mixed effects logistic regression models examining the likelihood of Medicaid-enrolled youth with mental illness having one or more dental visits during 2015. Model 1 includes all youth and excludes health condition and prospective risk variables (*n* = 6564). Model 2 excludes youth with no medical or pharmacy claims data and includes all explanatory variables (*n* = 5805)

Factors	Model 1			Model 2		
	Odds Ratio	***p***-value	Average Predicted Probability	Odds Ratio	***p***-value	Average Predicted Probability
	(95% CI)		(95% CI)	(95% CI)		(95% CI)
Sex						
Female	1.00 (Reference)		0.32 (0.30–0.33)	1.00 (Reference)		0.36 (0.32–0.36)
Male	0.82 (0.73–0.92)	0.001	0.28 (0.26–0.29)	0.78 (0.69–0.89)	< 0.001	0.31 (0.27–0.31)
Age						
7–10	1.00 (Reference)		0.4 (0.36–0.44)	1.00 (Reference)		0.45 (0.37–0.45)
11–14	0.75 (0.62–0.91)	0.004	0.34 (0.31–0.36)	0.76 (0.62–0.94)	0.010	0.38 (0.32–0.38)
15–18	0.59 (0.49–0.71)	< 0.001	0.29 (0.27–0.31)	am (0.53–0.79)	< 0.001	0.33 (0.29–0.33)
19–20	0.36 (0.29–0.41)	< 0.001	0.2 (0.18–0.22)	0.42 (0.33–0.54)	< 0.001	0.26 (0.20–0.26)
Race/Ethnicity						
White	1.00 (Reference)		0.28 (0.25–0.30)	1.00 (Reference)		0.32 (0.27–0.32)
Black /African American	1.05 (0.89–1.22)	0.580	0.28 (0.26–0.31)	1.07 (0.91–1.26)	0.428	0.33 (0.28–0.33)
Hispanic	1.25 (1.04–1.51)	0.015	0.32 (0.29–0.35)	1.32 (1.09–1.60)	0.005	0.38 (0.32–0.38)
Other	1.1 (0.77–1.55)	0.611	0.29 (0.23–0.36)	1.14 (0.79–1.65)	0.481	0.39 (0.24–0.39)
Not Provided	1.22 (0.96–1.55)	0.101	0.32 (0.28–0.35)	1.26 (0.98–1.61)	0.072	0.38 (0.30–0.38)
Language						
English	1.00 (Reference)		0.29 (0.28–0.30)	1.00 (Reference)		0.33 (0.29–0.33)
Not English	1.38 (1.13–1.68)	0.002	0.36 (0.31–0.40)	1.4 (1.14–1.73)	0.001	0.43 (0.34–0.43)
Education Levels in County						
15% + of adults have HS degree	1.00 (Ref)		0.31 (0.29–0.33)	1.00 (Reference)		0.35 (0.31–0.35)
< 15% of adults have HS degree	0.8 (0.68–0.93)	0.004	0.27 (0.24–0.29)	0.79 (0.67–0.93)	0.005	0.31 (0.26–0.31)
Medicaid Eligibility Group						
SSI	1.00 (Reference)		0.29 (0.26–0.32)	1.00 (Reference)		0.32 (0.26–0.32)
TANF	1.05 (0.87–1.28)	0.604	0.3 (0.28–0.32)	1.2 (0.97–1.47)	0.089	0.35 (0.31–0.35)
Other (Prim Child Welf)	1.05 (0.73–1.52)	0.773	0.3 (0.23–0.37)	1.14 (0.78–1.65)	0.497	0.39 (0.25–0.39)
NCHS Urban-Rural Category						
Large Central Metro (Most Urban)	1.00 (Reference)		0.29 (0.27–0.31)	1.00 (Reference)		0.33 (0.29–0.33)
Large Fringe Metro	1.16 (0.99–1.35)	0.064	0.32 (0.29–0.34)	1.17 (1.00–1.38)	0.050	0.37 (0.31–0.37)
Medium Metro	0.97 (0.81–1.17)	0.773	0.28 (0.25–0.31)	0.97 (0.80–1.18)	0.780	0.33 (0.27–0.33)
Small Metro	1.13 (0.76–1.66)	0.546	0.31 (0.24–0.39)	1.15 (0.76–1.74)	0.493	0.42 (0.25–0.42)
Non- Metro (Most Rural)	1.52 (0.98–2.36)	0.060	0.37 (0.28–0.47)	1.52 (0.96–2.41)	0.077	0.5 (0.30–0.50)
County is PCP HPSA						
No	1.00 (Reference)		0.3 (0.28–0.31)	1.00 (Reference)		0.33 (0.30–0.33)
Yes	0.63 (0.35–1.12)	0.112	0.21 (0.12–0.31)	0.69 (0.39–1. 25)	0.222	0.35 (0.14–0.35)
County is MH HPSA						
No	1.00 (Reference)		0.3 (0.28–0.31)	1.00 (Reference)		0.33 (0.30–0.33)
Yes	1 (0.77–1.28)	0.979	0.3 (0.25–0.34)	0.97 (0.75–1.27)	0.849	0.36 (0.26–0.36)
Did Member Have any Non-Dental Claims?						
No	1.00 (Ref)		0.14 (0.11–0.16)			
Yes	3.06 (2.44–3.84)	< 0.001	0.32 (0.30–0.33)			
ADHD						
No				1.00 (Reference)		0.32 (0.28–0.32)
Yes				1.34 (1.15–1.55)	< 0.001	0.39 (0.33–0.39)
Depression						
No				1.00 (Reference)		0.33 (0.30–0.33)
Yes				1.02 (0.88–1.18)	0.831	0.35 (0.29–0.35)
Bipolar						
No				1.00 (Reference)		0.33
						(0.30–0.33)
Yes				0.97 (0.82–1.16)	0.759	0.35
						(0.28–0.35)
Schizophrenia						
No				1.00 (Reference)		0.33 (0.30–0.33)
Yes				0.83 (0.62–1.11)	0.199	0.33 (0.23–0.34)
PTSD						
No				1.00 (Reference)		0.33 (0.30–0.33)
Yes				1.31 (0.97–1.75)	0.075	0.44 (0.31–0.44)
Anxiety, no PTSD						
No				1.00 (Reference)		0.33 (0.30–0.33)
Yes				1.15 (0.97–1.37)	0.111	0.38 (0.31–0.38)
Asthma						
No				1.00 (Reference)		0.33 (0.30–0.33)
Yes				1.16 (1.01–1.35)	0.041	0.37 (0.31–0.37)
Diabetes						
No				1.00 (Reference)		0.33 (0.30–0.33)
Yes				1.12 (0.73–1.73)	0.598	0.43 (0.25–0.43)
Hypertension						
No				1.00 (Reference)		0.33 (0.30–0.33)
Yes				1.06 (0.89–1.26)	0.526	0.36 (0.29–0.36)
Pregnancy						
No				1.00 (Reference)		0.34 (0.31–0.34)
Yes				0.61 (0.45–0.83)	0.002	0.28 (0.18–0.28)
Prospective Risk Score						
*(Continuous)*				1.09 (1.03–1.16)	0.004	See Fig. 1
Substance Use Disorder						
No				1.00 (Reference)		0.34 (0.31–0.34)
Yes				0.87 (0.72–1.06)	0.159	0.33 (0.26–0.33)

*Abbreviations*: *ADHD* Attention deficit/hyperactivity disorder, *HPSA* Health professional shortage area, *HS* High school, *NCHS* National Center for Health Statistics, *MH* Mental Health, *PCP* Primary care provider, *PTSD* Post-traumatic stress disorder, *SSI* Supplemental Security Income, *TANF* Temporary Aid for Needy Families

The only MI associated with statistically significant variation in both unadjusted and adjusted analyses was ADHD; youth with ADHD had higher odds of a dental visit than those without. Of comorbid physical health conditions, asthma was associated with significantly greater odds of dental visits in both sets of analyses while pregnancy was associated with significantly lower odds of dental visits in both sets of analyses. Further, again based on statistical significance in both unadjusted and adjusted analyses, being male was associated with lower odds of dental visits relative to femaleness, there were decreasing odds of having one or more dental visits with increasing age, Hispanic persons had greater odds of a dental visit than non-Hispanic whites, Non-English speakers had higher odds of having dental visits compared to English speakers, and living in a county where less than 15% of adults were high school graduates was associated with lower odds of a dental visit relative to those in counties with more educated adults. In both sets of analyses there was a statistically significant association between prospective risk score and dental visits; as risk score increased the likelihood of dental visits increased.

While PTSD, hypertension, and substance use disorder were associated with statistically significant variations in the likelihood of dental visits based on unadjusted analyses (Table 1), these variables were non-significant in the adjusted model (Table 2). In post hoc analyses we found that substance use disorders were significantly associated with age; that is, older youth were more likely to have substance use disorder diagnoses than younger youth (*p* < 0.05). Consequently, when we stratified the unadjusted analyses of dental visits and substance use disorders by age the associations were non-significant (*p* > 0.05; data not shown). Neither unadjusted nor adjusted analyses identified statistically significant variation in dental visits associated with Medicaid eligibility group, NCHS urban-rural category, PCP HPSA, MH HPSA, depression, bipolar, schizophrenia, anxiety, or diabetes.

## Discussion

We found that Medicaid-enrolled youth with MI had low rates of dental care visits. Overall, 29.0% of Medicaid-enrolled young persons with MI received at least one dental visit during 2015. In contrast, between 38.8 and 45.7% (age-adjusted) of young persons in general population Medicaid plans in Florida had at least one dental visit for the same year [21]. Our findings are in contrast to previous research indicating that children with special healthcare needs (CSHCN) are more likely to receive preventive dental care than those without such needs [22]; however, that study was not limited to Medicaid enrollees. Further, it is difficult to draw meaningful comparisons between our findings regarding youth with MI and studies on CSHCN given the broad definition of CSHCN [23] and thus the heterogeneity of that population. Our results align with past studies that found that Medicaid-enrolled children who qualified for Medicaid due to disability were less likely to have dental visits than those qualified based on income [13].

Recognizing the wide range of health needs and priorities of youth with MI, it may be speculated that care for MI is being prioritized over dental visits [13]. Similar prioritization behavior was observed in another study examining the non-dental healthcare utilization of enrollees in the specialty health plan that provided data for this study, all of whom had MI. That research found that, upon enrollment in the plan, enrollees’ average time elapsed to first visit to a behavioral health provider was shorter than the average time to first visit to a physical health provider [16], suggesting that mental health needs may have been viewed as more critical than routine visits or care for physical health conditions. Thus, there may be opportunities to improve dental utilization in this population through cross-sector referral relationships and interprofessional care, including programs that partner dental providers with community-based mental health providers.

The low rate of dental service use in the population of youth with MI is a concern. Although there is little research about the impact of MI on oral health among children and adolescents, MI in adults is associated with an increased risk of dental decay [4]. Potential explanations for the higher rates of caries in the adult MI population include poor oral hygiene and side effects of medications [1–3]. Antidepressants, mood stabilizers, and antipsychotics can have oral manifestations such as hypofunction of salivary glands, which can result in reduced salivary flow and increase the risk of dental caries [5]. The use of psychotropic medications in young persons is increasing [24], so youth with MI may be increasingly at risk of dental caries due to the oral health-related side effects of these medications.

### Mental and physical health diagnoses

While there was a low rate of dental service use in youth with MI overall, particular MIs (i.e., anxiety, depression, bipolar disorder, and schizophrenia) were not generally associated with an increased or decreased likelihood of having a dental visit in Medicaid-enrolled youth with MIs. There was one exception – having an ADHD diagnosis was associated with higher odds of dental visits relative to not having this diagnosis. It is possible that this association is due to a heightened need for treatment in youth with ADHD. Recent systematic reviews of research on ADHD and oral health suggest that ADHD is associated with both traumatic dental injuries and dental caries, and ADHD may be associated with poor oral hygiene [25, 26]. It is of note that the youth in our study with ADHD also had a history of another MI; ADHD diagnoses alone would not qualify them for enrollment in the Medicaid specialty plan that supplied data (see “Data Source”). Thus, future studies are warranted to explore the impact of ADHD on the oral health and dental visits of youth with and without other MIs to better understand if interactions or additive relationships exist.

Some comorbid physical health conditions experienced by youth with MI were also associated with dental visits. Notably, asthma was associated with a greater likelihood of dental visits. These findings mirror those seen with ADHD. While asthma and ADHD are very different conditions, the medications often used to treat these conditions are associated with many negative oral health outcomes [27].. For both conditions, an increased risk of dental caries has been linked to medication side-effects. For example, inhaler medications, used to treat asthma, may reduce salivary flow and pH [28] and methylphenidate, used to treat ADHD, has been associated with reduction in salivary flow leading to xerostomia [29]. While our data disallow causal statements, it may be that these associations were due to an increased need for treatment-based dental visits.

Another physical health condition associated with dental care was pregnancy; young persons with MI and a recent or current pregnancy had lower odds of having dental visits than those without. These findings are consistent with previous research in the general population indicating that many women do not receive dental care during pregnancy [30]. While the nature of the association between oral health and negative birth outcomes such as preeclampsia and low birthweight is unclear and research examining this association has yielded inconsistent results [31–35], changes in oral health status can occur during pregnancy [36]. Accordingly, dental visits during pregnancy are safe and are recommended by professional organizations including the American Academy of Pediatric Dentistry [37] and the American Association of Obstetricians and Gynecologists [38]. There may be a need for programs focused on facilitating dental visits among pregnant or recently pregnant Medicaid-enrolled adolescents with MI. As there are HEDIS measures focused on prenatal and postpartum care, it may be operationally efficient for Medicaid plans working towards increasing HEDIS rates to develop programmatic strategies which jointly target pregnancy-related care and dental care in pregnant youth with MI.

### Predicted need for future healthcare: prospective risk

Medicaid plans might also leverage risk scores when developing or enhancing programs to reach youth with MI who are unlikely to have a dental visit. We found that as the prospective risk scores of children with MI increased, their likelihood of having a dental visit also increased, and vice versa (see Fig. 1). These scores are often used by health plans to stratify enrollees into or within population management, disease management, or complex case management programs, and persons with high risk receive more intensive services [39]. Our results suggest, however, that youth with MIs and low risk scores were less likely to have dental visits relative to those with higher scores. We also found that children with MI but no claims for non-dental healthcare services were markedly less likely to have a dental visit relative to those who had one or more non-dental healthcare services. Consequently, youth with MI who might be deprioritized for case management based on risk scores or minimal prior utilization might conversely need to be a priority for dental-focused programs.

### Socio-demographic characteristics

#### Age

Numerous demographic characteristics were also associated with dental visits in youth with MI. We found that the likelihood of dental visits varied by age, with 7 to 10-year-old children having higher odds of having visits compared to older youth, and the odds of visits decreased with age through the pre-teen and adolescent years. This is consistent with patterns observed in general population Medicaid managed care plans in Florida [21], as well as findings based on nationally representative samples of youth regardless of insurance coverage type [40, 41]. Older children and adolescents have distinct needs for dental care, including a high rate of caries [41]. Our findings suggest that, in particular, older youth with MI who are enrolled in Medicaid may need to be targeted in outreach efforts to facilitate dental visits.

#### Education

Education at the county level appeared to play a role in dental utilization in young Medicaid enrollees with MI. Those living in counties where less than 15% of adults have a high school degree were less likely to have dental visits than those in counties with higher levels of education. While ours was not a direct measure of parental education, this finding was consistent with research that found that children of parents without high school degrees were far less likely to have a dental visit than children with parents who are college graduates [42]. These results may point to the need for health plans and providers to use health literacy-informed strategies when communicating with parents and older youth about the dental services that are covered by Medicaid as well as the importance of dental care.

#### Ethnicity and language

Ethnicity played a two-part role in this study; Hispanic youth with MI had higher odds of dental visits than non-Hispanic white youth with MI, and those whose primary language was not English were more likely to have a dental visit than English-speakers. Our findings contrast with past research suggesting that children in non-English speaking households face barriers to dental care [43], but geographic differences in the dental workforce may explain our results. Our data are from Florida, a traditional destination state for Spanish-speaking immigrants. Consequently, there is an increased demand for and supply of Spanish speaking health care providers compared to other areas of the US [44]. This is especially true in counties like Miami-Dade, where over 60% of its residents speak Spanish at home and over a third speak English less than “Very Well” [45]. In total, 29.4% of Florida dentists speak Spanish [46].

Florida has more Hispanic dentists relative to other parts of the country [46], and Hispanic persons may prefer ethnically similar dentists [47]. Thus, while we are unable to attribute causality, the relative abundance of Hispanic dentists in Florida might explain our finding that Hispanic youth with MI had higher odds of dental visits as compared to non-Hispanic white youth with MI. Variations in states’ dental workforces might also explain past studies’ findings that the impact of race and ethnicity on dental care differs by state [13, 48]. Together, these study results underscore the potential importance of linguistic and ethnic diversity in health plans’ dental provider networks.

### Limitations and future research opportunities

Limitations of our study included the cross-sectional, observational nature of our data which disallowed us from making causal attributions. Additionally, the study was exploratory; our findings are data-driven. Nevertheless, we identified important associations and patterns that will guide future hypothesis-driven research. Further, although claims data are a rich source of information about diagnosed health conditions, conditions are only reflected in these data if they are associated with rendered healthcare services. As a result, undiagnosed conditions and conditions not associated with services were not examined. We found that 11.6% of youth in our study had no claims for non-dental services during the period studied, although all had a history of MI. Consequently, MIs and comorbid medical conditions for this subset of young persons were unknown. Given the eligibility criteria for the health plan (see Data Source & Study Population) it is likely that these persons had a MI that we were unable to identify with the available data, but a minority may not have had MI. Specifically, persons with a prior pharmacy claim for a medication used to treat major depression, bipolar disorder, obsessive-compulsive disorder, schizophrenia, or another psychotic or delusional disorder were qualified for inclusion in the plan even if no diagnosis was present. Off-label use of these medications may have resulted in enrollment of some persons without MI conditions, and such persons may be included in our analysis if they or a guardian did not choose to change health plans and consequently remained enrolled throughout 2015. We are unable to determine which, if any, of the youth in our analysis may have been misidentified, but we can feel confident that most did have MI based on the population’s high use of mental health treatment (particularly inpatient psychiatric treatment) and high rates of juvenile justice involvement and involuntary examinations for mental illness [16].

Similarly, our data do not contain information about some barriers to care that youth in our study may have experienced. We could not assess knowledge about dental benefits or the importance of dental care. Additionally, both in Florida and nationally, a lack of available dentists has impeded dental service use by Medicaid enrollees, and we were unable to examine whether availability was an issue for the young persons in our study. While access to dental care was reportedly a strength of the Medicaid plan that provided data for this study [16], a statewide shortage of Medicaid-participating dental providers was identified in Florida’s 2015 Roadmap for Oral Health. Further, lack of transportation has been a common barrier to the receipt of dental services by Medicaid enrollees and our data did not provide insight into transportation challenges. These issues are not unique to our study; such limitations would be true of any study using claims data to examine healthcare utilization, including past studies examining dental visits in Medicaid-enrolled children [13]. Even so, these are important barriers to care, so future studies are needed to examine these challenges in Medicaid-enrolled youth with MI.

While our results provide important information about young people during the developmental periods in which MI becomes increasingly prevalent and serious MI begins to emerge [9], future studies should examine dental visits in younger children with pediatric MI or serious emotional disturbance. Additionally, states’ Medicaid coverage varies for persons ages 19 and 20 years. While Florida has not expanded Medicaid under the Affordable Care Act, many other states have expanded access to Medicaid for persons in this age group. Consequently, analyses of 19 and 20-year-old Medicaid enrollees in expansion states would include persons with relatively higher income and potentially different diagnostic profiles and utilization patterns than enrollees in Florida.

With our use of Florida Medicaid data, generalizability must be considered. While all state Medicaid plans are federally mandated to cover dental services for children and adolescents enrolled in Medicaid, utilization patterns may vary due to state-specific differences in the administration of Medicaid dental benefits or differing reimbursement policies and rates. In addition, we examined data from one specialty health plan that only enrolled persons with serious MI. Although the plan that provided data for the current study was the first such plan, similar “vertical carve-out” Medicaid plans are becoming increasingly common [14, 15]. Our study provides useful information about the characteristics associated with dental service utilization within the vulnerable population of youth covered by this relatively new type of Medicaid managed care plan, but future studies are warranted to confirm that our findings generalize to other specialty plans serving youth with MI. Additionally, more research is needed to determine the extent to which our observed rates of dental utilization are related to features of the health plan itself versus the characteristics of the population served by the plan.

Another opportunity to build on the current study is to expand on the HEDIS-defined measure of dental visits when examining the dental care of Medicaid-enrolled youth with MI. The HEDIS measure does not examine preventive and treatment visits separately, it only includes youth continuously enrolled in a health plan for a calendar year, and it only measures care financed by the plan and provided by licensed dentists or dental hygienists. Thus, although children and adolescents may receive dental care from non-dental providers or in school-based programs that do not bill Medicaid, our study does not include such services. Nevertheless, the HEDIS definition of annual dental visits is a widely-used, standard, validated measure. As such, our results can confidently be compared to health plans within the same state for the same year [21]. Further, results can be compared to national annual Medicaid managed care rates over time, as data for this measure have been collected by NCQA since 2002 for the age groups examined in the current paper [17].

### Contributions

Our study provides timely information about a vulnerable subpopulation of Medicaid enrollees – youth with mental health conditions. The Centers for Medicare and Medicaid Services (CMS) has been engaged in an ongoing Children’s Oral Health Initiative which aims to increase dental service use in Medicaid-enrolled youth; consequently, there have been marked increases in their receipt of dental care [17]. Even so, there remains significant variations in dental care within that population [13]. Past research suggests that increasing reimbursements for dentists, elevating use of and payments for dental hygienists, and providing clearer definitions of coverage may increase dental provider participation in Medicaid and thus increase utilization [49, 50]. The current study builds on this past research, and results from our sizable sample of youth with MI can guide practices to increase dental service use in these vulnerable Medicaid enrollees. We found that Medicaid-enrolled children and adolescents with MI have low rates of dental visits, while also identifying specific, actionable opportunities for improvement within this population.

## Conclusions

Overall, children and adolescents with MI had a relatively low likelihood of receiving annual dental visits, but we identified significant variations in dental service use within this population. Given the great importance of oral health to the well-being of young people and the fact that dental decay, periodontal disease, and tooth loss are common in persons with mental health conditions [3, 4], it is critical to facilitate dental care in children and adolescents with MI. In addition to providing unique insights that may be used to develop hypotheses for future studies, our results can be used to develop interventions focused on subpopulations of children and adolescents with MI who have a particularly low likelihood of using dental services. State Medicaid agencies, Medicaid managed care programs, dental benefit management organizations, and population health management programs can leverage our findings as they develop targeted strategies to increase dental visits in – and consequently improve the overall current and long-term wellbeing of – the vulnerable population of young people with MI.

## Data Availability

The data that support the findings of this study are available from Magellan Health, Inc., on behalf of the State of Florida, Agency for Health Care Administration, but restrictions apply to the availability of these data. The data were collected and analyzed during the administration and delivery of Medicaid health benefits and thus contain personal healthcare information about Florida Medicaid enrollees. By statute, such data are not publicly available. Deidentified, HIPAA-compliant data could be generated by the Magellan authors and provided to qualified researchers upon reasonable request and with permission of the State of Florida, Agency for Health Care Administration.
